# Sustained 10-year gain in adult life expectancy following antiretroviral therapy roll-out in rural Malawi: July 2005 to June 2014

**DOI:** 10.1093/ije/dyw208

**Published:** 2016-10-14

**Authors:** Alison J Price, Judith Glynn, Menard Chihana, Ndoliwe Kayuni, Sian Floyd, Emma Slaymaker, Georges Reniers, Basia Zaba, Estelle McLean, Fredrick Kalobekamo, Olivier Koole, Moffat Nyirenda, Amelia C Crampin

**Affiliations:** 1 Department of Infectious Disease Epidemiology, Faculty of Epidemiology and Population Health, London School of Hygiene and Tropical Medicine, London, UK; 2 Department of Population Health, Faculty of Epidemiology and Population Health, London School of Hygiene and Tropical Medicine and; 3 Karonga Prevention Study, Chilumba, Karonga, Malawi

**Keywords:** HIV, ART, mortality, life-expectancy, Malawi, sub-Saharan Africa

## Abstract

**Background:** Improved life expectancy in high HIV prevalence populations has been observed since antiretroviral therapy (ART) scale-up. However, it is unclear if the benefits are sustained, and the mortality among HIV-positive individuals not (yet) on ART is not well described. We assessed temporal change in mortality over 9 years in rural Malawi.

**Methods:** Within a demographic surveillance site in northern rural Malawi, we combined demographic, HIV and ART uptake data. We calculated life expectancy using Kaplan-Meier estimates, and compared mortality rates and rate ratios using Poisson regression, by period of ART availability (July 2005–June 2008, July 2008–June 2011 and July 2011–June 2014).

**Results:** Among 32 664 individuals there were 1424 deaths; 1930 individuals were known HIV-positive, of whom 1382 started ART. Overall, life expectancy at age 15 years increased by 10 years within 5 years of ART introduction, and plateaued. Age-standardized adult mortality rates declined from 11.3/1000 to 7.5/1000 person-years between the first and last time period. In July 2011-June 2014 compared with July 2005–June 2008, mortality declined in HIV-positive individuals on ART (rate ratio adjusted (aRR) for age, sex, location and education, 0.3; 95% confidence interval (CI) 0.2–0.5) and in those not (yet) on ART (aRR 0.3; 95%CI 0.1–0.5) but not in HIV-negative individuals (aRR 1.1; 95%CI 0.7–1.9).

**Conclusions:** Total population adult life expectancy increased toward that of HIV-negative individuals by 2011 and remained raised. The reduction in all-cause and HIV-related mortality in HIV-positive individuals not (yet) on ART suggests ART uptake is occurring at an earlier disease stage, particularly in women.

## Introduction

In 2004, Malawi initiated a public health approach to HIV care and treatment services. The programme aimed to deliver antiretroviral therapy (ART) to all eligible HIV-positive individuals presenting to decentralized clinics, with minimal reliance on laboratory support for determining eligibility and for monitoring patients in care.^1^^,^^2^ Early success of this initiative was evident within the first year of scale-up, with increased survival among those started on treatment^3^ and declines in all-cause mortality rates at the population level.^4^

Since the scale-up of ART programmes, substantial reductions in adult mortality ^5^ and improved life expectancy among HIV-positive individuals on ART^6–9^ have been observed in other high HIV prevalence sub-Saharan African (SSA) countries. However, the trends in all-cause and cause-specific mortality patterns in HIV-positive individuals who are not (yet) receiving ART are not well described. Understanding the temporal trends in these different groups is essential for evaluating the impact of future test and treat strategies and establishing whether continued improvement in mortality might be expected in proportion to the increased burden of ART care.^10^^,^^11^

Although introduced nationally in 2004, ART was not available free at the point of care in northern rural Malawi until July 2005. Within a year, all-cause mortality had declined by 16%^4^ and within the next 2 years, mortality declined by 32%.^12^ In this population, deaths attributable to HIV in adults, as assigned by verbal autopsy, decreased from 42% in 2005 to 17% in 2009.^13^ It is not clear if these early improvements in mortality are sustained beyond this time, during a period of increasingly decentralized ART care, and where there is little availability of CD4 cell counts or viral load monitoring for those on long-term treatment.

This study uses population-level demographic and HIV test data from northern rural Malawi to investigate the effect of the ART programme on life expectancy and adult all-cause and cause-specific mortality, including HIV-positive individuals with and without ART uptake, covering a 9-year period of scale-up of ART availability through increasingly decentralized care.

## Methods

### Setting

The Karonga demographic surveillance site (DSS), was established in 2002 in a rural population of nearly 33 000 individuals in northern Malawi, and conducts continuous demographic surveillance, with rigorous identification procedures permitting linkage to data collected in other studies nested within the surveillance population.^14^

HIV counselling and testing (HCT) of the DSS population has been conducted for various studies since 1985. In 2005–06, HIV prevalence was estimated at 11.5%^15^ from a population-representative stratified sample sero-survey in those aged 18 to 59 years. Four annual house-to-house cross-sectional HIV sero-surveys were conducted between September 2007 and September 2011, for all individuals aged 15 years or older, involving counselling, enquiry about previous HIV testing and ART uptake start date, and rapid HIV testing with results available to participants. By 2010, HIV prevalence had declined to 8%.^16^ In 2013, an additional survey invited participants aged 18 years and older who had missed earlier rounds to screen for HIV infection, using rapid testing with results immediately available.

ART became available in Karonga District Hospital, 70 km north of the DSS area, in July 2005. Within the DSS, ART was first available from a single rural hospital in 2006,^12^ then from an additional health centre in 2010 and then from three further health centres between 2011 and early 2012. In the wider Karonga district, there were four ART clinics in 2008, six clinics in 2010 and 16 clinics by the end of 2012.^17^ From 2005 to 2010, individuals were eligible for ART if they were in World Health Organization (WHO) clinical stage 3 or 4 or had a CD4 cell count < 250 cells/mm. This was extended to CD4 < 350 cells/mm^3^ in 2011 (and to < 500 cells/mm^3^ in July 2014, after this study period). Throughout the period of this study, CD4 cell counts were not consistently available and viral load monitoring was not available in the surveillance area. Before and during ART roll-out there was a policy to provide preventative care to all HIV-positive patients, including nutritional support, TB screening and treatment and prophylactic cotrimoxazole antibiotics, although delivery was variable.

By mid-2008, ART uptake in the DSS area was estimated to be at least 60% of those eligible, with greater uptake (65%) in women than in men (48%).^18^ In the district, 6-month retention in care increased from 70% in the first 2 years of ART availability to 92% in 2011–12.^17^

### Data sources

Information relating to an individual’s first ART start date was collected through interviews and linked ART records. If conflicting data were observed between clinic register and self-reported ART start dates, the clinic-register date was used. Individual-level data on pre-ART care was not available for this analysis. HIV test results were obtained from testing conducted in population-level and clinic-based studies between 2005 and 2014. Socio-demographic data, including location of residence (< 1 km, ≥ 1 km to tarmac road) and highest level of education (none/primary incomplete, primary complete, secondary incomplete, secondary complete/tertiary) were obtained from the annual census and continuous demographic surveillance.

Cause of death (HV-related, non-HIV-related) was ascertained from verbal autopsy data. The verbal autopsies are conducted following all deaths in the DSS, by medical assistants with additional training using a semi-structured questionnaire adapted from the standard World Health Organization questionnaire. Whenever possible, the informant is a close relative of the deceased and nursed them through their final illness, and the information is collected approximately 2–4 weeks after the death. Each verbal autopsy questionnaire is independently reviewed by two clinicians, and in the event of discrepancies a third reviewer assigns the cause of death after viewing the initial two reviews. Data on HIV status may be available to the reviewer within the verbal autopsy questionnaire or through access to the HIV test database, if the deceased had participated in a study providing HIV testing. If a decision cannot be made on the cause, the death is coded as un-specifiable. Of all adult deaths, 9% were either unspecified or unknown.

### Statistical analysis

The analysis was restricted to adults aged 15 years or older. To assess life expectancy trends, individuals contributed exposure time during their residence in the DSS from January 2003 (the start of demographic surveillance in the area, including a period preceding population-level HIV testing and ART availability), or their date of in-migration, if later, until the earliest of 31 December 2013, death or out-migration. Adult life expectancy was computed as the area under the Kaplan-Meier survival curve for each calendar year. It can be interpreted as the number of additional years that an adult– here defined as 15 years old– can expect to live under the age- and sex- specific mortality rates that prevail in a particular year.

For all-cause and cause-specific mortality analyses, individuals contributed exposure time during their residence in the DSS from the start of July 2005 (when ART was first available in the district and population-representative HIV test data were available in the DSS), or their date of in-migration if later, until the earliest of 30 June 2014, death or out-migration. Returning and repeat migrants only contributed person-years while resident in the DSS area. HIV status and ART treatment status were treated as time-varying covariates. Person-years were classified as HIV-negative up to 3 years after the latest HIV-negative test unless there was an earlier positive test. Person-years were classified as HIV-positive after a positive test. All other periods (before the first HIV test and > 3 years after the latest negative test) were classified as HIV status unknown, to avoid selection bias.

For individuals who reported ART initiation at a clinic outside the district before July 2005, only person-years from study entry at 1 July 2005 contributed to this analysis. ART uptake in HIV-positive individuals was classified according to ever use and duration of treatment. Individuals were categorized as ‘ever started on ART’ from the first ART start date and as ‘not (yet) on ART’ for the period between an HIV-positive test result and an ART start date, if one existed, or to study exit. In those who ever started ART, individuals were also categorized by duration of antiretroviral treatment, with early treatment defined as less than 6 months on ART (as this has been shown to be a high-risk period^19^^,^^20^^)^ and longer-term treatment defined as 6 months or longer.

Age- and sex-specific mortality rates were calculated for three periods of ART availability/scaling-up; early (July 2005 to June 2008), mid (July 2008 to June 2011) and late (July 2011 to June 2014), and using age categories 15–24, 25–34, 35–44, 45–54, 55–64 and 65 years or more. To account for changing age structure in the population, age-standardized mortality rates were calculated by using the total population during the follow-up period as the standard population.

All-cause and cause-specific (HIV-related, non-HIV-related) mortality rate ratios were calculated by period of ART availability using Poisson regression. To examine the role of factors that might affect the association between period of ART availability and risk for all-cause and cause-specific mortality, participants were grouped according to sex, HIV infection and ART uptake, location of residence, age group and highest attained level of education, with adjustment for these factors where appropriate. Likelihood ratio χ^2^ tests were used to test for difference in the risk for all-cause and cause-specific deaths in adults over the three calendar periods. Tests for heterogeneity were calculated by entering a term for the interaction between the time period and the co-factor variable in the logistic regression models, and the statistical significance of the interaction terms were calculated with likelihood ratio tests.

These demographic data represent an open cohort, with mortality outcomes available for all those remaining resident in the DSS area. To understand better the extent to which different population sub-groups (who may be at higher risk for death) are associated with risk for departure from the DSS, we conducted an exploratory analysis. All calculations used Stata version 14.0 (Stata Corporation, College Station, TX).

Ethics approval for the studies was granted by the National Health Sciences Research Committee of Malawi (NHSR #419, #424, #448, #968 and #1072) and the Ethics Committee of the London School of Hygiene & Tropical Medicine (#5081, #5067, #5214, #6126 and #6303).

## Results

After excluding 11 individuals with indeterminate HIV test results or a single negative test after an earlier positive result, and 26 individuals with an ART start date but a later HIV-negative test result, 14 845 men and 17 819 women aged 15 years and older contributed a total of 161 849 person years and 1424 deaths between July 2005 and June 2014. Of 1930 individuals identified as HIV-positive during follow-up, ART uptake was established for 1382 individuals: 1192 (86%) via a clinic register link and 190 (14%) via self-report. Data on WHO stage at ART initiation were available for 97% of those with a clinic register link: initiation at WHO stage four declined from 33% in July 2005–June 2008, to 8% in July 2008–June 2011 and then to 7% in July 2011–June 2014 (data not shown in table).

**Figure 1.**
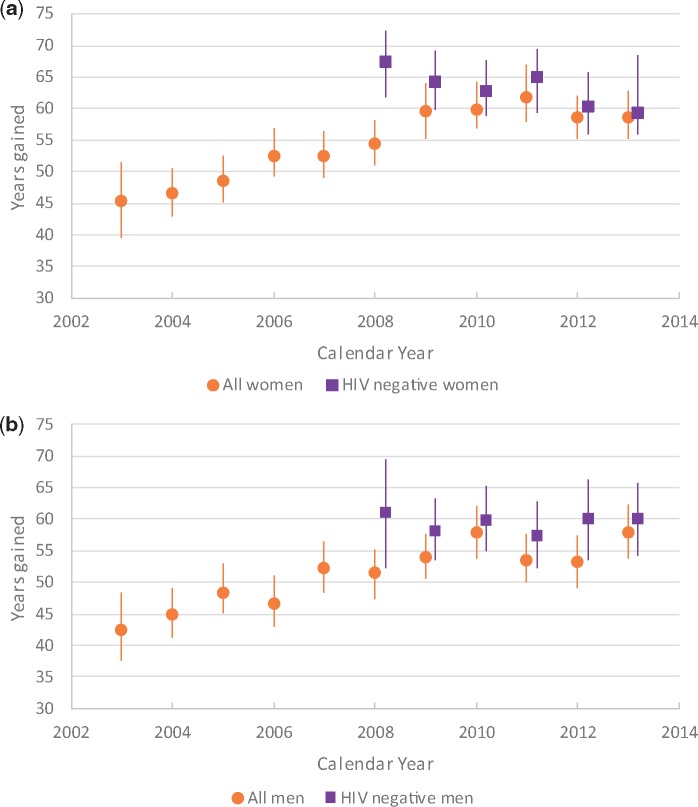
Life expectancy at 15 years in women (a) and men (b): 2003-2013.

All-cause adult age-standardized mortality rates declined from 11.3/1000 person-years (py) in July 2005–June 2008, to 10.7/1000 py in July 2008–June 2011 and then to 7.5/1000 py in July 2011–June 2014. Overall life expectancy at age 15 increased from 42.5 (95% CI 37.5–48.4) to 57.9 years (95% CI 53.8–62.1) in men between 2003 and 2010 and from 45.5 (95% CI 39.6–51.5) to 61.8 (95% CI 58.0–66.9) in women between 2003 and 2011, and then plateaued: attaining life expectancy comparable to that observed in HIV-negative men and women (Figure 1a and b).

Age-specific mortality rates and rate ratios in men and women in the three periods of ART availability by sex and 10-year age groups are shown in Table 1. In men and women, mortality was lowest in those aged 15–24 years and highest in those aged 65 years and older, with no evidence for change over time. A large reduction in mortality was observed in women aged 25 to 44 years and men aged 25 to 54 years between the early and mid-period of ART availability. In the late period, these mortality reductions were sustained in women and men aged 25 to 44 years but not in men aged 45 to 54 years.

**Table 1.** Age specific mortality rates for adults 15 years and older by sex and period of ART availability

	Person- years	Deaths	Rate/1000 py	95% CI	Crude mortality rate ratio	95% CI	Adjusted mortality rate ratio^a^	95% CI	*P*-value^b^
Male, age, years									
15–24									
Jul 05–Jun 08	8940	14	1.6	0.9–2.6	1.0		1.0		
Jul 08–Jun 11	8910	14	1.6	0.9–2.7	1.0	0.5–2.1	1.1	0.5–2.2	
Jul 11–Jun 14	9968	18	1.8	1.1–2.9	1.2	0.6–2.3	1.2	0.6–2.3	0.9
25–34									
Jul 05–Jun 08	6220	46	7.4	5.5–9.9	1.0		1.0		
Jul 08–Jun 11	6316	24	3.8	2.5–5.7	0.5	0.3–0.8	0.5	0.3–0.9	
Jul 11–Jun 14	6534	25	3.8	2.6–5.7	0.5	0.3–0.8	0.5	0.3–0.9	0.01
35–44									
Jul 05–Jun 08	3521	58	16.5	12.7–21.3	1.0		1		
Jul 08–Jun 11	3968	33	8.3	5.9–11.7	0.5	0.3–0.8	0.5	0.3–0.8	
Jul 11–Jun 14	4520	47	10.4	7.8–13.8	0.6	0.4–0.9	0.6	0.4–0.9	0.004
45–54									
Jul 05–Jun 08	2006	41	20.4	15.1–27.8	1.0		1.0		
Jul 08–Jun 11	2311	19	8.2	5.2–12.9	0.4	0.2–0.7	0.4	0.2–0.7	
Jul 11–Jun 14	2630	35	13.3	9.6–18.5	0.7	0.4–1.0	0.7	0.4–1.1	0.004
55–64									
Jul 05–Jun 08	1319	32	24.3	17.2–34.3	1.0		1.0		
Jul 08–Jun 11	1347	27	20.0	13.7–29.2	0.8	0.5–1.4	0.9	0.5–1.4	
Jul 11–Jun 14	1475	19	12.9	8.2–20.2	0.5	0.3–0.9	0.6	0.3–1.0	0.1
65+									
Jul 05–Jun 08	1698	75	44.2	35.2–55.4	1.0		1.0		
Jul 08–Jun 11	1739	94	54.0	44.1–66.1	1.2	0.9–1.7	1.3	1.0–1.8	
Jul 11–Jun 14	1855	75	40.4	32.2–50.7	0.9	0.7–1.3	1.0	0.7–1.4	0.2
Female, age, years									
15–24									
Jul 05–Jun 08	9356	13	1.4	0.8–2.4	1.0		1.0		
Jul 08–Jun 11	9477	15	1.6	1.0–2.6	1.1	0.5–2.4	1.3	0.6–2.8	
Jul 11–Jun 14	10698	18	1.7	1.1–2.7	1.2	0.6–2.5	1.4	0.7–2.9	0.6
25–34									
Jul 05–Jun 08	6894	64	9.3	7.3–11.9	1.0		1		
Jul 08–Jun 11	7298	23	3.2	2.1–4.7	0.3	0.2–0.5	0.3	0.2–0.6	
Jul 11–Jun 14	7802	21	2.7	1.8–4.1	0.3	0.2–0.5	0.3	0.2–0.5	< 0.001
35–44									
Jul 05–Jun 08	3933	42	10.7	7.9–14.5	1.0		1.0		
Jul 08–Jun 11	4359	24	5.5	3.7–8.2	0.5	0.3–0.9	0.5	0.3–0.9	
Jul 11–Jun 14	5156	19	3.7	2.4–5.8	0.3	0.2–0.6	0.4	0.2–0.6	0.001
45–54									
Jul 05–Jun 08	2640	30	11.4	7.9–16.3	1.0		1.0		
Jul 08–Jun 11	2860	22	7.7	5.1–11.7	0.7	0.4–1.2	0.7	0.4–1.2	
Jul 11–Jun 14	3149	24	7.6	5.1–11.4	0.7	0.4–1.1	0.7	0.4–1.2	0.3
55–64									
Jul 05–Jun 08	1791	34	19.0	13.6–26.6	1.0		1.0		
Jul 08–Jun 11	1929	20	10.4	6.7–16.1	0.5	0.3–0.9	0.6	0.3–1.0	
Jul 11–Jun 14	2043	22	10.8	7.1–16.4	0.6	0.3–1.0	0.6	0.3–1.0	0.1
65+									
Jul 05–Jun 08	2254	118	52.4	43.7–62.7	1.0		1.0		
Jul 08–Jun 11	2342	102	43.5	35.9–52.9	0.8	0.6–1.1	0.8	0.6–1.1	
Jul 11–Jun 14	2591	117	45.2	37.7–54.1	0.9	0.7–1.1	0.9	0.7–1.1	0.4

py, person-years; June, June; Jul, July; -, range.

^a^Adjustment made for location of residence (< 1 km, ≥ 1 km from roadside), education (none/incomplete primary, complete primary, incomplete secondary, complete secondary/tertiary, unknown), where appropriate.

^b^*P*-value test for difference in the association between time-period of ART availability (July 2005–June 2008, July 2008–June 2011 and July 2011–June 2014) and mortality, calculated with a likelihood ratio test.

All-cause crude mortality rates and rate ratios adjusted for sex, age group, location of residence and education are shown in Table 2. After adjusting for other factors there was a 30% reduction in mortality in the adult population between the early and mid-period of ART availability, which was sustained during the late period. Mortality rates were similar in men and women in the early period but a greater reduction was observed in women (30%) than men (20%), although there was no evidence for heterogeneity in the association by sex (*P*-heterogeneity = 0.4). The fall was sustained in both sexes in the late period (July 2011 to June 2014).

**Table 2.** All-cause mortality for adults aged 15 years and older by period of ART availability and HIV infection/ART use

	Person-years	Deaths	Rate/1000 py	95% CI	Crude mortality rate ratio	95% CI	Adjusted mortality rate ratio^a^	95% CI	Adjusted mortality rate ratio^b^	95% CI	*P*-value^c^
Overall population											
Period of ART											
Jul 05–Jun 08	50571	567	11.2	10.3–12.2	1.0		1.0		1.0		
Jul 08–Jun 11	52857	417	7.9	7.2–8.7	0.7	0.6–0.8	0.7	0.6–0.8	0.7	0.6–0.8	
Jul 11–Jun 14	58421	440	7.5	6.9–8.3	0.7	0.6–0.8	0.7	0.6–0.8	0.7	0.6–0.8	< 0.001
Sex by period of ART											
Male											
Jul 05–Jun 08	23703	266	11.2	10.0–12.7	1.0		1.0		1.0		
Jul 08–Jun 11	245915	211	8.6	7.5–9.8	0.8	0.6–0.9	0.8	0.6–0.9	0.8	0.7–0.9	
Jul 11–Jun 14	269812	219	8.1	7.1–9.3	0.7	0.6–0.9	0.7	0.6–0.9	0.8	0.6–0.9	0.004
Female											
Jul 05–Jun 08	26868	301	11.2	10.0–12.5	1.0		1.0		1.0		
Jul 08–Jun 11	28266	206	7.3	6.4–8.4	0.7	0.5–0.8	0.6	0.5–0.8	0.7	0.6–0.8	
Jul 11–Jun 14	31439	221	7.0	6.2–8.0	0.6	0.5–0.7	0.6	0.5–0.7	0.7	0.5–0.8	< 0.001
HIV infection/ART use by period of ART											
HIV-negative											
Jul 05–Jun 08	4876	17	3.5	2.2–5.6	1.0		1.0		1.0		
Jul 08–Jun 11	35080	173	4.9	4.2–5.7	1.4	0.9–2.3	1.1	0.7–1.8	1.1	0.7–1.8	
Jul 11–Jun 14	32700	162	5.0	4.2–5.8	1.4	0.9–2.3	1.1	0.7–1.8	1.1	0.7–1.9	0.9
HIV-positive; ever on ART											
Jul 05–Jun 08	531	37	69.7	50.5–96.1	1.0		1.0		1.0		
Jul 08–Jun 11	1771	63	35.6	27.8–45.5	0.5	0.3–0.8	0.5	0.3–0.7	0.5	0.3–0.7	
Jul 11–Jun 14	2710	60	22.1	17.2–28.5	0.3	0.2–0.5	0.3	0.2–0.5	0.3	0.2–0.5	< 0.001
HIV-positive; not (yet) on ART											
Jul 05–Jun 08	390	20	51.3	33.1–79.5	1.0		1.0		1.0		
Jul 08–Jun 11	1484	18	12.1	7.6–19.3	0.2	0.1–0.4	0.2	0.1–0.4	0.2	0.1–0.4	
Jul 11–Jun 14	1064	17	16.0	10.0–25.7	0.3	0.2–0.6	0.3	0.1–0.5	0.3	0.1–0.5	< 0.001
HIV status unknown											
Jul 05–Jun 08	44742	493	11.0	10.1–12.0	1.0		1.0		1.0		
Jul 08–Jun 11	14523	163	11.2	9.6–13.1	1.0	0.9–1.2	0.9	0.8–1.1	1.0	0.8–1.1	
Jul 11–Jun 14	21947	201	9.2	8.0–10.5	0.8	0.7–1.0	0.8	0.7–1.0	0.9	0.7,–1.0	0.2
HIV-positive ever on ART^d^											
HIV-positive; < 6 months on treatment											
Jul 05–Jun 08	160	24	149.4	100.2–222.9	1.0		1.0		1.0		
Jul 08–Jun 11	238	25	105.2	71.8–155.7	0.7	0.4–1.2	0.7	0.4–1.2	0.7	0.4–1.2	
Jul 11–Jun 14	154	8	52.0	26.0–103.9	0.3	0.2–0.8	0.3	0.2–0.8	0.3	0.1–0.7	0.01
HIV-positive; ≥ 6 months on treatment											
Jul 05–Jun 08	371	13	35.1	20.4–60.4	1.0		1.0		1.0		
Jul 08–Jun 11	1533	38	24.8	18.0–34.1	0.7	0.4–1.3	0.7	0.3–1.2	0.7	0.3–1.2	
Jul 11–Jun 14	2586	52	20.3	15.5–26.7	0.6	0.3–1.1	0.5	0.3–1.0	0.5	0.3–1.0	0.20
HIV-positive; not (yet) on ART											
Male											
Jul 05–Jun 08	146	7	48.1	22.9–100.9	1.0		1.0		1.0		
Jul 08–Jun 11	571	10	17.5	9.4–32.6	0.4	0.1–1.0	0.4	0.1–0.9	0.40	0.1–1.0	
Jul 11–Jun 14	458	9	19.6	10.2–37.8	0.4	0.2–1.1	0.4	0.1–1.0	0.40	0.1–1.0	0.13
Female											
Jul 05–Jun 08	244	13	53.3	30.9–91.7	1.0		1.0		1.0		
Jul 08–Jun 11	913	8	8.8	4.4–17.5	0.2	0.1–0.4	0.1	0.1–0.4	0.2	0.1–0.4	
Jul 11–Jun 14	606	8	13.2	6.6–26.4	0.2	0.1–0.6	0.2	0.1–0.5	0.2	0.1–0.6	< 0.001
HIV-positive; not (yet) on ART											
Location of residence < 1 km from tarmac											
Jul 05–Jun 08	216	15	69.5	41.9–115.2	1.0		1.0		1.0		
Jul 08–Jun 11	779	6	7.7	3.5–17.1	0.1	0.04–0.3	0.1	0.04–0.3	0.1	0.04–0.3	
Jul 11–Jun 14	593	8	13.5	6.7–27.0	0.2	0.1–0.5	0.2	0.1–0.4	0.2	0.1–0.4	< 0.001
Location of residence > 1 km from tarmac											
Jul 05–Jun 08	171	5	28.8	12.0–69.1	1.0		1.0		1.0		
Jul 08–Jun 11	674	12	17.0	9.7–30.0	0.6	0.2–1.7	0.5	0.2–1.5	0.5	0.2–1.6	
Jul 11–Jun 14	456	9	19.1	9.9–36.7	0.7	0.2–2.0	0.5	0.2–1.6	0.6	0.2–1.7	0.50

Jun, June; Jul, July; py, person-years; -, range.

^a^Adjustment made for age (15–24, 25–34, 35–44, 45–54, 55–64, 65+ years), sex (male, female), where appropriate.

^b^Adjustment made for age (15–24, 25–34, 35–44, 45–54, 55–64, 65+ years), sex (male, female), location of residence (< 1 km, ≥ 1 km from roadside), education (none/incomplete primary, complete primary, incomplete secondary, complete secondary/tertiary, unknown), where appropriate.

^c^*P*-value test for difference in the association between period of ART availability (July 2005–June 2008, July 2008–June 2011 and July 2011–June 2014) and mortality, calculated with a likelihood ratio test.

^d^In those who used ART during follow-up, the data are presented by duration of treatment (< 6 and ≥ 6 months).

Large reductions in mortality were observed in HIV-positive individuals on ART, with crude mortality (CMR) falling from 69.7/1000 py in July 2005–June 2008 to 35.6/1000 py in July 2008–June 2011 to 22.1/1000 py in July 2011–June 2014. A large fall in mortality was observed in HIV-positive individuals not (yet) on ART between the early and middle period (CMR 51.3 vs 12.1/1000 py; aRR 0.2; 95% CI: 0.1–0.4) that was sustained in the late period. Among those who started ART, mortality in the early stages of ART treatment (< 6 months) declined over time from 149.4/1000 py in the early period, to 105.2/1000 py in the middle period and then to 52.0/1000 py in the late period (*P* = 0.01). There was no evidence for a decline in mortality in those on longer-term treatment (> 6months) (*P* = 0.2), or in HIV-negative (*P* = 0.9) or HIV-unknown individuals (*P* = 0.2) over the study period, although mortality remained lowest in HIV-negative individuals.

**Figure 2.**
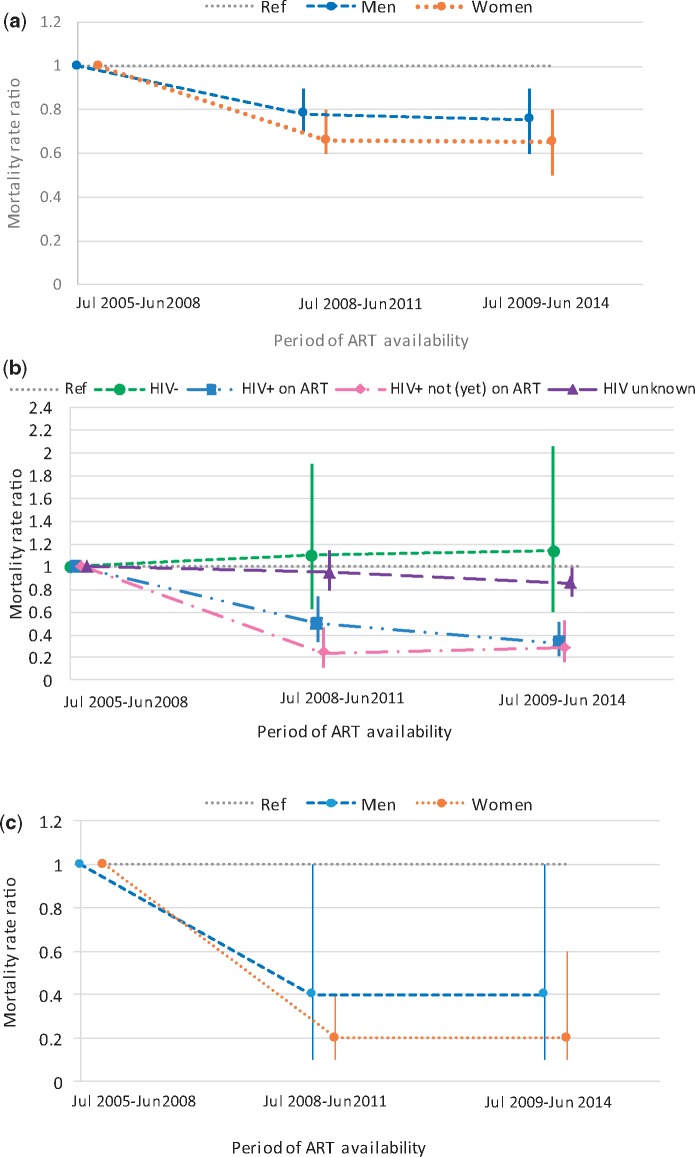
Adult mortality rate ratios of the association between period of ART availability and (a) sex; (b) HIV infection and ART use, and; (c) sex in those not (yet) on ART.

In HIV-positive individuals not (yet) on ART, mortality was higher in women than men in the early period of ART availability and greater mortality reductions were observed in women than in men (53.3 to 13.2 per 1000 py in women vs 48.1 to 19.6 per 1000 py in men), although there was no evidence that the association differed by sex (*P*-heterogeneity = 0.5). Mortality was higher among HIV-positive individuals not on ART and living close to a tarmac road during the early period of ART availability compared with those living further away; however, those close to the tarmac road experienced a greater decline in mortality between the early and late period than those living further away (69.5 to 13.5/1000 py vs 28.8 to 19.1/1000 py (Table 2). Figure 2a-c shows declining mortality in men and women in the total adult population (Figure 2a) and in HIV-positive individuals (Figure 2b), including men and women not (yet) on ART (Figure 2c).

Table 3 shows that HIV-related mortality in adults declined by 70% between the early and late period of ART availability (CMR 3.4 vs 1.2/1000 py; aRR 0.3; 95% CI: 0. 2–0.4), evident in both men and women (*P* < 0.001). Mortality due to HIV-related causes declined progressively in HIV-positive individuals on treatment, with a 70% lower rate of HIV-related deaths in the late ART period compared with the early period (58.4 vs 17.0 per 1000 py; *P* < 0.001). Large declines in HIV-related deaths were also observed in HIV-positive individuals not (yet) on ART (70%). In those who ever started ART, early mortality (< 6 months on treatment) due to HIV-related causes dropped from 130.8/1000 py to 54.3/1000 py and then to 45.5/1000 py over the three time periods (*P* = 0.02).

**Table 3.** HIV-related mortality for adults 15 years and older by period of ART availability and HIV infection/ART use

	Person- years	HIV- related deaths	Rate/1000 py	95% CI	Crude mortality rate ratio	95% CI	Adjusted mortality rate ratio^a^	95% CI	Adjusted mortality rate ratio^b^	95% CI	*P*-value^c^
Overall population											
Period of ART											
Jul 05–Jun 08	50571	174	3.4	3.0–4.0	1.0		1.0		1.0		
Jul 08–Jun 11	52857	73	1.4	1.1–1.7	0.4	0.3–0 .5	0.4	0.3–0.5	0.4	0.3–0.5	
Jul 11–Jun 14	58421	69	1.2	0.9–1.5	0.3	0.3–0.5	0.3	0.2–0.4	0.3	0.2–0.4	< 0.001
Sex											
Male											
Jul 05–Jun 08	23703	91	3.8	3.1–4.7	1.0		1.0		1.0		
Jul 08–Jun 11	24592	37	1.5	1.1–2.1	0.4	0.3–0.6	0.4	0.3–0.5	0.4	0.3–0.5	
Jul 11–Jun 14	26981	35	1.3	0.9–1.8	0.3	0.2–0.5	0.3	0.2–0.5	0.3	0.2–0.5	< 0.001
Female											
Jul 05–Jun 08	26868	83	3.1	2.5–3.8	1.0		1.0		1.0		
Jul 08–Jun 11	28266	36	1.3	0.9–1.8	0.4	0.3–0.6	0.4	0.3–0.6	0.4	0.3–0.6	
Jul 11–Dec13	31439	34	1.1	0.8–1.5	0.4	0.2–0.5	0.3	0.2–0.5	0.3	0.2–0.5	< 0.001
HIV infection/ART use by period of ART											
HIV-positive; ever on ART											
Jul 05–Jun 08	531	31	58.4	41.0–83.0	1.0		1.0		1.0		
Jul 08–Jun 11	1771	51	38.8	21.9–37.9	0.5	0.3–0.8	0.5	0.3–0.7	0.5	0.3–0.7	
Jul 11–Jun 14	2710	46	17.0	12.7–22.7	0.3	0.2–0.5	0.3	0.2–0.4	0.3	0.2–0.4	< 0.001
HIV-positive; not (yet) on ART											
Jul 05–Jun 08	390	12	30.8	17.5–54.2	1.0		1.0		1.0		
Jul 08–Jun 11	1485	9	6.1	3.2–11.7	0.2	0.1– 0.5	0.2	0.1–0.5	0.2	0.1–0.5	
Jul 11–Jun 14	1064	11	10.3	5.7–18.7	0.3	0.1– 0.8	0.3	0.1–0.7	0.3	0.1–0.7	0.001
HIV status unknown											
Jul 05–Jun 08	44742	131	2.9	2.5–3.5	1.0		1.0		1.0		
Jul 08–Jun 11	14523	12	0.8	0.5–1.5	0.3	0.2–0.5	0.3	0.2–0.5	0.3	0.1–0.5	
Jul 11–Jun 14	21947	12	0.5	0.3–1.0	0.2	0.1–0.3	0.2	0.1–0.4	0.2	0.1–0.3	< 0.001
HIV-positive ever on ART^d^											
HIV test positive; < 6 months on treatment											
Jul 05–Jun 08	161	21	130.8	85.3–200.5	1.0		1.0		1.0		
Jul 08–Jun 11	238	20	54.3	54.3–130.5	0.6	0.3–1.2	0.6	0.3–1.1	0.6	0.3–1.2	
Jul 11–Jun 14	154	7	45.5	20.7–95.4	0.3	0.1–0.8	0.3	0.1–0.8	0.3	0.1–0.7	0.02
HIV test positive; ≥ 6 months on treatment											
Jul 05–Jun 08	371	10	27.0	14.5–50.2	1.0		1.0		1.0		
Jul 08–Jun 11	1533	31	20.2	14.2–28.7	0.7	0.4–1.5	0.7	0.4–1.5	0.70	0.4–1.5	
Jul 11–Jun 14	2556	39	15.3	11.1–20.9	0.6	0.3–1.1	0.5	0.3–1.1	0.50	0.3–1.1	0.2

Jun, June; Jul, July; py, person-years; -, range.

^a^Adjustment made for age (15–24, 25–34, 35–44, 45–54, 55–64, 65+ years), sex (male, female), where appropriate.

^b^Adjustment made for age (15–24, 25–34, 35–44, 45–54, 55–64, 65+ years), sex (male, female), location of residence (< 1 km, ≥ 1 km from roadside), education (none/incomplete primary, complete primary, incomplete secondary, complete secondary/tertiary, unknown), where appropriate.

^c^*P*-value test for difference in the association between period of ART availability (July 2005–June 2008, July 2008–June 2011 and July 2011–June 2014) and mortality, calculated with a likelihood ratio test.

^d^In those who used ART during follow-up, the data are presented by duration of treatment (< 6 and ≥ 6 months).

In this adult population there was no evidence for a decline in non-HIV-related mortality between the early and late time periods of ART availability (Table 4; 6.7 vs 5.6/1000 py; aRR 0.9; 95% CI 0. 8–1.0; *P* = 0.2), and with no evidence for difference between men and women (*P*-heterogeneity = 0.4). Between the early and late periods, a large drop in non-HIV-related mortality was observed in HIV-positive individuals not (yet) on treatment (18.0 vs 3.8 per 1000 py; aRR 0.2; 95% CI 0.1–0.6; *P* = 0.02) that was not seen in HIV-positive individuals on ART, HIV-negative or HIV-unknown individuals.

**Table 4.** Non-HIV-related mortality for adults 15 years and older by period of ART availability and HIV infection/ART use

	Person-years	Non-HIV-related deaths	Rate/1000 py	95% CI	Crude mortality rate ratio	95% CI	Adjusted mortality rate ratio^a^	95% CI	Adjusted mortality rate ratio^b^	95% CI	*P*-value^c^
Overall population											
Period of ART											
Jul 05–Jun 08	50571	341	6.7	6.1–7.5	1.0		1.0		1.0		
Jul 08–Jun 11	52857	313	5.9	5.3–6.6‐	0.9	0.8–1.0	0.9	0.8–1.0	0.9	0.8–1.1	
Jul 11–Jun 14	58421	325	5.6	5.0–6.2‐	0.8	0.7–1.0	0.8	0.7–1.0	0.9	0.8–1.0	0.2
Sex											
Male											
Jul 05–Jun 08	23703	154	6.5	5.5–7.6‐	1.0		1.0		1.0		
Jul 08–Jun 11	24592	158	6.4	5.5–7.5‐	1.0	0.8–1.2	1.0	0.8–1.2	1.03	0.8–1.3	
Jul 11–Jun 14	26981	163	6.0	5.2–7.0‐	0.9	0.7–1.2	0.9	0.7–1.2	1.00	0.8–1.2	1.00
Female											
Jul 05–Jun 08	26868	187	7.0	6.0–8.0‐	1.0		1.0		1.0		
Jul 08–Jun 11	28266	155	5.5	4.7–6.4‐	0.8	0.6–1.0	0.8	0.6–1.0	0.8	0.7–1.0	
Jul 11–Jun 14	31439	162	5.2	4.4–6.0‐	0.7	0.6–0.9	0.7	0.6–0.9	0.8	0.6–1.0	0.04
HIV infection/ART use by period of ART											
HIV-negative	4876	15	3.1	1.9–5.1‐	1.0		1.0		1.0		
Jul 05–Jun 08	35080	157	4.5	3.8–5.2‐	1.5	0.9–2.5	1.1	0.7–1.9	1.1	0.7–1.9	
Jul 08–Jun 11	32700	149	4.6	3.9–5.4	1.5	0.9–2.5	1.2	0.7–2.0	1.2	0.7–2.0	0.8
Jul 11–Jun 14											
HIV-positive; ever on ART											
Jul 05–Jun 08	531	6	11.3	5.1–25.1	1.0		1.0		1.0		
Jul 08–Jun 11	1771	11	6.2	3.4–11.2	0.5	0.2–1.5	0.5	0.2–1.3	0.5	0.2–1.3	
Jul 11–Jun 14	2710	12	4.4	2.5–7.8	0.4	0.1–1.0	0.4	0.1–1.0	0.3	0.1–0.9	0.1
HIV-positive; not (yet) on ART											
Jul 05–Jun 08	390	7	18.0	8.6–37.7‐	1.0		1.0		1.0		
Jul 08–Jun 11	1484	9	6.1	3.2–11.7	0.3	0.1–0.9	0.3	0.1–0.8	0.3	0.1–0.8	
Jul 11–Jun 14	1064	4	3.8	1.4–10.0	0.2	0.1–0.7	0.2	0.1– 0.6	0.2	0.1–0.6	0.02
HIV status unknown											
Jul 05–Jun 08	44774	313	7.0	6.3–7.8‐	1.0		1.0		1.0		
Jul 08–Jun 11	14523	136	9.4	7.9–11.1	1.3	1.1–1.6	1.2	1.0–1.5	1.2	1.0–1.5	
Jul 11–Jun 14	21947	160	7.3	6.2–8.5‐	1.0	0.9–1.3	1.0	0.8–1.2	1.1	0.9–1.3	0.10
HIV-positive ever on ART^d^											
HIV test positive; < 6 months on treatment											
Jul 05–Jun 08	161	3	18.7	6.0–54.5‐	1.0		1.0		1.0		
Jul 08–Jun 11	238	4	16.8	6.4–45.3	0.9	0.2–4.0	0.9	0.2–3.9	0.9	0.2–4.1	
Jul 11–Jun 14	154	1	6.5	1.0–50.4‐	0.3	0.04–3.3	0.3	0.04–3.3	0.3	0.04–3.3	0.6
HIV test positive; ≥ 6 months on treatment											
Jul 05–Jun 08	371	3	8.1	2.6–25.1	1.0		1.0		1.0		
Jul 08–Jun 11	1533	7	4.6	2.2–9.6	0.6	0.1–2.2	0.4	0.1–1.7	0.4	0.1–1.7	
Jul 11–Jun 14	2556	11	4.3	2.4–7.8	0.5	0.1–1.9	0.4	0.1–1.6	0.4	0.1–1.5	0.5

Jun, June; Jul, July; py, person-years; -, range.

^a^Adjustment made for age (15–24, 25–34, 35–44, 45–54, 55–64, 65+ years), sex (male, female), where appropriate.

^b^Adjustment made for age (15–24, 25–34, 35–44, 45–54, 55–64, 65+ years), sex (male, female), location of residence (< 1 km, ≥ 1 km from roadside), education (none/incomplete primary, complete primary, incomplete secondary, complete secondary/tertiary, unknown), where appropriate.

^c^*P*-value test for difference in the association between period of ART availability (July 2005–June 2008, July 2008–June 2011 and July 2011–June 2014) and mortality, calculated with a likelihood ratio test.

^c^In those who used ART during follow-up, the data are presented by duration of treatment (< 6 and ≥ 6 months).

Out-migration from the study population was associated with unknown HIV status (aRR HIV unknown vs HIV-negative 2.0; 95% CI 1.9–2.1); with being younger (aRR 15–24 vs 65 years or older 8.1; 95% CI 7.2–9.2); with female sex (aRR female vs male 1.2; 95% CI 1.2–1.3), and; with higher educational attainment (aRR secondary vs none/incomplete primary 4.2; 95% CI 3.6–4.8; results not shown in a table), after controlling for potential confounders.

## Discussion

Our data show that total population adult life expectancy at age 15 in rural northern Malawi increased by almost 10 years between 2005 and 2011 and then plateaued, reaching the life expectancy estimate of HIV-negative individuals. Our results are of a similar or greater magnitude to the increases in mean length of life observed elsewhere in high HIV prevalence sub Saharan Africa where national ART programmes exist.^7^^,^^21^ In our population, the observed increase in adult life expectancy is largely a result of the decline in adult mortality of HIV-positive individuals during a 9-year period of ART scale-up, decentralized care and annual household-level HIV testing, with almost universal acceptance of test results.^16^^,^^22^

The large reductions in all-cause and HIV-related mortality over time in those not (yet) on ART indicates a shift in ART initiation, from late-stage AIDS to an earlier phase of infection, getting HIV-positive individuals onto treatment early, leaving only relatively healthy positive individuals without (or yet to start) treatment. The sustained reduction in mortality over time in HIV-positive individuals and reduction in HIV-related mortality are consistent with our earlier findings^13^ and confirm that the early success of the ART programme^12^ has continued over the longer term in this rural, resource-constrained setting. However, our results suggest that smaller gains are being made in men than in women, particularly among those who are HIV-positive and yet to start ART. These findings indicate that men with a positive HIV diagnosis are remaining, to a greater extent than women, without treatment until too late into the disease: a finding consistent with other studies from sub-Saharan Africa.^23^ In our setting, the late treatment occurred even though the individuals were aware of their HIV status, an issue that may be important to consider as the global impetus for achieving 90‐90‐90 targets intensifies, requiring initiation of ART immediately after testing HIV positive.^10^^,^^11^

Among HIV-positive individuals, the initially high mortality in the first 6 months of treatment more than halved over time as individuals moved onto treatment at an earlier stage of disease (evidenced by 7% initiating ART in WHO stage 4 in the late phase, compared with 33% in the early phase); but it remained a high-risk period, as observed elsewhere.^24^ The large decline in HIV-related deaths in those of unknown HIV status suggests that few undiagnosed HIV-positive individuals remain in the surveillance population, following high participation rates in multiple HIV sero-surveys, during which 95% received their results.^16^ Access difficulties, including distance to clinics and transport costs, are established barriers for linkage to and retention in care.^25^ In Malawi, decentralization of ART services is associated with improved retention of patients in care.^17^ However, lower declines in all-cause and HIV-related mortality were observed in those living in more rural areas compared with those living close to a tarmac road, which may suggest more unmet need peripherally despite decentralization.

Our study linked annual sero-survey HIV test results and ART clinic data from across the surveillance population, which facilitated categorization of individuals according to HIV status and ART uptake to investigate in detail the impact of ART on mortality over time. However, our data include a relatively small HIV-positive adult population, so the confidence intervals on our mortality estimates are wide, particularly for age-group categories and other sub-group analyses. Nonetheless, HIV testing was provided in the community to a population representative sample between 2005 and 2006 and across all adults (15 years and older) between 2007 and 2011, so selection bias should not be a major concern for our study. HIV-unknown individuals contributed the largest proportion of person-years in the early phase of ART availability but, since HIV testing across the entire population did not start until 2007, the unknown population is likely to be representative of the overall population. Inclusion of undiagnosed, late-stage HIV-positive individuals in the HIV-unknown group will have contributed to the higher HIV-related mortality in the early period compared with the late period, when fewer chronically undiagnosed HIV-positive individuals remain in the population. Adults of unknown HIV status, young individuals, women and those with higher educational attainment were most at risk of out-migration from the DSS. Data on the health status of out-migrants at the time of departure (or their subsequent survival or death) were not available, so the extent to which their departure affected our mortality and life expectancy estimates could not be determined. However, in other settings where tracking of out-migrants has been implemented, higher mortality in those individuals has been reported,^(^^26^^)^ so it is possible that our results (and those from other DSS and open cohort studies) somewhat underestimate mortality and overestimate life expectancy gains. It is also possible that compared with the general population, higher life expectancy gains were observed in our surveillance population where health-related research has been conducted for many years. We have previously shown that during this time period, an increasing proportion of verbal autopsy informants reported that they knew the deceased’s HIV status, and the HIV status recorded on the verbal autopsy report influences the way the clinician reviewer assigns cause of death irrespective of symptoms.^27^ Hence it is possible that our findings underestimate to some extent the true gains in HIV-related mortality although, given the stable estimates of non-HIV-related mortality, misclassification is unlikely to be a major issue.

As the numbers of infected individuals surviving on ART increase; long-term retention and adherence; onward transmission from those who have interrupted treatment; and, identification of virological failure in long-term ART users, will be a challenge for the current public health programme and require ongoing monitoring and evaluation. In 2011, Malawi expanded the criteria for ART eligibility to include HIV-positive individuals with CD4 counts < 350 cell/mm^3^, and initiation of life-long treatment in pregnant and breastfeeding women irrespective of disease severity,^28^ and to HIV-positive individuals with CD4 counts < 500/mm^3^ from July 2014 onwards.^29^ These changes facilitate earlier initiation and increase the numbers of individuals alive on ART, but the sustained gains in adult life expectancy depend on continued access to effective treatment and high levels of retention and adherence of those in care. With a growing number of individuals surviving on ART, early identification of virological failure with the option to switch treatment at decentralized clinics is needed but will be a challenge in this setting

### Conclusions

Our results suggest that large reductions in adult mortality and increasing adult life expectancy have been achieved in rural -Malawi over a 9-year period of ART availability, as HIV infected individuals move into care at an earlier stage of disease. HIV-positive men may be more likely than women to remain without ART until a late stage of disease. These data confirm that the success achieved in the early phase of ART availability has continued, highlighting the success of the public health approach to HIV care in Malawi. Improvement in mortality was observed in those who have accessed care and also in those not yet in care.

## Funding

This work was supported by a Wellcome Trust Award (096249/Z/11/A).

**Conflict of interest**: None to declare.

**Key Messages**

- A sustained increased adult life expectancy during a period of decentralized ART care in Malawi.
- A decline in all-cause and HIV-related mortality in HIV-positive adults on and not (yet) on ART.
- Suggests earlier uptake of ART in women than in men.
- Success of a public health approach to ART provision in Malawi.
